# Investigating the Effectiveness of Technology-Based Distal Interventions for Postpartum Depression and Anxiety: Systematic Review and Meta-Analysis

**DOI:** 10.2196/53236

**Published:** 2024-11-19

**Authors:** Sarah P Brocklehurst, Alyssa R Morse, Tegan Cruwys, Philip J Batterham, Liana Leach, Alysia M Robertson, Aseel Sahib, Colette T Burke, Jessica Nguyen, Alison L Calear

**Affiliations:** 1 School of Medicine and Psychology The Australian National University Canberra Australia; 2 Centre for Mental Health Research The Australian National University Canberra Australia; 3 National Centre for Epidemiology and Population Health The Australian National University Canberra Australia

**Keywords:** postpartum, depression, anxiety, birth, adoptive, parents, mobile phone

## Abstract

**Background:**

Postpartum anxiety and depression are common in new parents. While effective interventions exist, they are often delivered in person, which can be a barrier for some parents seeking help. One approach to overcoming these barriers is the delivery of evidence-based self-help interventions via websites, smartphone apps, and other digital media.

**Objective:**

This study aims to evaluate the effectiveness of technology-based distal interventions in reducing or preventing symptoms of postpartum depression or anxiety in male and female birth and adoptive parents, explore the effectiveness of technology-based distal interventions in increasing social ties, and determine the level of adherence to and satisfaction with technology-based distal interventions.

**Methods:**

A systematic review and series of meta-analyses were conducted. Three electronic bibliographic databases (PsycINFO, PubMed, and Cochrane Library) were searched for randomized controlled trials evaluating technology-based distal interventions for postpartum depression or anxiety in birth and adoptive parents. Searches were updated on August 1, 2023, before conducting the final meta-analyses. Data on trial characteristics, effectiveness, adherence, satisfaction, and quality were extracted. Screening and data extraction were conducted by 2 reviewers. Risk of bias was assessed using the Joanna Briggs Institute quality rating scale for randomized controlled trials. Studies were initially synthesized qualitatively. Where possible, studies were also quantitatively synthesized through 5 meta-analyses.

**Results:**

Overall, 18 articles met the inclusion criteria for the systematic review, with 14 (78%) providing sufficient data for a meta-analysis. A small significant between-group effect on depression favored the intervention conditions at the postintervention (Cohen *d*=–0.28, 95% CI –0.41 to –0.15; *P*<.001) and follow-up (Cohen *d*=–0.27, 95% CI –0.52 to –0.02; *P*=.03) time points. A small significant effect on anxiety also favored the intervention conditions at the postintervention time point (Cohen *d*=–0.29, 95% CI –0.48 to –0.10; *P*=.002), with a medium effect at follow-up (Cohen *d*=–0.47, 95% CI –0.88 to –0.05; *P*=.03). The effect on social ties was not significant at the postintervention time point (Cohen *d*=0.04, 95% CI –0.12 to 0.21; *P*=.61). Effective interventions tended to be web-based cognitive behavioral therapy programs with reminders. Adherence varied considerably between studies, whereas satisfaction tended to be high for most studies.

**Conclusions:**

Technology-based distal interventions are effective in reducing symptoms of postpartum depression and anxiety in birth mothers. Key limitations of the reviewed evidence include heterogeneity in outcome measures, studies being underpowered to detect modest effects, and the exclusion of key populations from the evidence base. More research needs to be conducted with birth fathers and adoptive parents to better ascertain the effectiveness of interventions in these populations, as well as to further assess the effect of technology-based distal interventions on social ties.

**Trial Registration:**

PROSPERO CRD42021290525; https://www.crd.york.ac.uk/prospero/display_record.php?RecordID=290525

## Introduction

### Background

Postpartum depression and anxiety can be experienced by any parent, including birth or adoptive mothers and fathers, as they welcome their new child into their family [1]. Postpartum depression and anxiety are defined in the *Diagnostic and Statistical Manual of Mental Disorders, Fifth Edition*, as a major depressive disorder or generalized anxiety disorder with postpartum onset [2]. In clinical settings and research, it is well accepted that symptom onset can occur up to 12 months after birth or adoption [3,4].

Postpartum depression is quite common. For birth parents, it is estimated that approximately 10% of fathers [5] and 13% of mothers [6] experience postpartum depression. For adoptive parents, it impacts approximately 11% of fathers [7] and 8.8% of mothers [6]. Postpartum anxiety is also relatively common, impacting approximately 4.4% to 10.8% of parents [8]. There are many risk factors that contribute to parents’ vulnerability to developing postpartum depression and anxiety symptoms. These include low self-esteem; low income; history of mood disorders; young age; a negative cognitive attributional style; and stressful life events, including marital strain, past miscarriage, and childhood sexual abuse [9]. New parents are also often at higher risk of social isolation due to fatigue and limited spare time [10]. This social isolation may negatively affect their mental health, exacerbating the chances of developing postpartum depression or anxiety. The development of anxiety and depression symptoms may also increase social withdrawal due to feelings of incompetence and worthlessness, creating a cycle in which poorer mental health and social isolation fuel each other [10].

Preventing and treating postpartum depression and anxiety is paramount as they can have significant short- and long-term effects on parents and children [11]. Well-evidenced short-term effects include sleep disturbance [12], poorer parent-infant attachment, and partner relationship dissatisfaction [11], whereas long-term effects can include poorer cognitive development for the infant [13], the breakdown of close relationships [11], and challenges in parental responsiveness to infant cues such as facial expressions [14].

There is a large body of research on interventions to prevent and treat postpartum depression and anxiety. This research provides support for a number of therapeutic approaches aimed at preventing and reducing symptoms, including cognitive behavioral therapy (CBT) [15] and interpersonal psychotherapy (IPT) [16]. In addition, some interventions have targeted social isolation as their mechanism of action to reduce postpartum depression and anxiety symptoms [17].

While effective interventions for postpartum depression and anxiety exist, they are often delivered in person or with the direct and immediate involvement of a therapist (eg, over videoconferencing, phone calls, or SMS text messaging). The need for client and therapist interaction to be in person or in real time creates barriers for many parents who need help. These barriers include not having the transport to get to a session, not having a babysitter or feeling uncomfortable leaving their child to go to the sessions or take a phone call, and the stigma attached to seeking help [18]. These barriers potentially reduce the number of parents receiving evidence-based treatments for their postpartum depression and anxiety [18]. In addition, professional mental health services are often overwhelmed, resulting in challenges obtaining an appointment for in-person therapy [19]. Therefore, it is important to identify and develop more cost- and resource-effective interventions to increase accessibility.

One approach to overcome these barriers is the delivery of evidence-based self-help via websites, smartphone apps, and chatbot interventions. These can be completed at a time and place most convenient for the user, reducing the need for travel or fitting appointments around feeding and sleeping schedules. Some of these interventions have been empirically tested, and there is evidence that these methods can be effective in reducing symptoms of postpartum anxiety and depression [20] and tend to be well accepted by users [21].

### This Research

The aims of this systematic review and meta-analysis were to identify and assess the effectiveness of technology-based distal interventions for postpartum depression and anxiety in male and female birth and adoptive parents. We defined distal interventions as those that are delivered remotely without the direct and immediate input of a therapist or support person. Distal interventions could include podcasts, mobile apps, automated chatbots, and self-help web-based programs. Technology-based interventions were defined as programs that are accessed or downloaded through the internet. The effectiveness of each intervention to reduce symptoms of postpartum depression and anxiety, as well as the adherence to and satisfaction with the intervention, was assessed. Given the important role of social isolation in the development of postpartum depression and anxiety, this review also aimed to explore whether technology-based distal interventions for postpartum depression and anxiety are effective in increasing social ties. Although multiple systematic reviews and meta-analyses have been conducted on technology-based interventions for postnatal anxiety and depression, they tend to only include birth mothers, and none focus on distal interventions [22-24]. Therefore, this is the first study to review the effectiveness of distal and technology-based interventions on birth and adoptive mothers and fathers.

## Methods

### Protocol and Registration

This review was prospectively registered with PROSPERO (CRD42021290525; December 10, 2021).

### Search Strategy

A comprehensive literature search was conducted in the following electronic bibliographic databases: PsycINFO, PubMed, and Cochrane Library. The search included a combination of five key blocks of terms related to the main objective of the review: (1) anxiety or depression (eg, depress* and anxi*), (2) postnatal (eg, perinatal and parent), (3) intervention (eg, program and cognitive therapy), (4) distal and technology based (eg, remote and web*site*), and (5) trial (eg, experiment and evaluation). Search terms were generated, trialed, and revised by the research team, with additional search terms identified from relevant research literature. Terms were entered in the appropriate search fields (eg, title, abstract, keywords or text words, and subject headings) and adapted to meet the requirements of each database. Multimedia Appendix 1 provides a full search strategy example, and a complete list of search terms can be found in the preregistration.

There were no publication date restrictions. Only English-language studies were included. One reviewer ran the search in all databases. The reference lists of the included studies and previous literature reviews in this field were hand searched. An exploratory search was conducted on November 30, 2021, and the searches were updated on April 17, 2023, and again on August 1, 2023, before conducting the final meta-analyses.

### Study Selection

Studies were considered for inclusion in the review and meta-analysis if (1) they were a randomized controlled trial (RCT) where an active intervention was compared to a treatment-as-usual, no intervention, waitlist, or attention control condition; (2) the evaluated intervention was primarily designed to reduce or prevent the depression or anxiety of parents of any gender, age, or nationality who were in the postpartum period (ie, first 12 months) following the birth of their child, including birth and adoptive mothers and fathers; (3) the intervention was delivered distally in the community without the direct and immediate input of a therapist or support person and was technology based (eg, self-help website, podcast, or mobile app; communication could be included if it was automated, infrequent [ie, not scheduled meetings], or optional [eg, SMS text message reminders]); (4) the intervention was self-guided but delivered therapeutic content (eg, CBT or psychoeducation); (5) the primary outcome of the study was the effect of the intervention on symptoms of anxiety or depression (collected via either validated self-report measures or clinical interview) at the postintervention and follow-up time points (if measured); and (6) the study was published in an English-language peer-reviewed journal. Noninferiority RCTs were excluded to ensure comparability of effect sizes. Gray literature, non–peer-reviewed journal articles, and book chapters were excluded, as were conference abstracts and proceedings, dissertations, editorials, viewpoints, perspectives, reviews, and commentaries.

### Data Extraction

This systematic review is reported in accordance with the PRISMA (Preferred Reporting Items for Systematic Reviews and Meta-Analyses) statement (Multimedia Appendix 2). The screening, risk-of-bias assessment, and data extraction were managed using the Covidence software (Veritas Health Innovation) [25].

The titles and abstracts of all studies were screened independently by the first author and a second reviewer to identify studies that potentially met the inclusion criteria. All reviewers were briefed and provided with the detailed review protocol before screening abstracts. The full texts of potentially eligible studies were retrieved and independently assessed for eligibility by the first author and a second reviewer in accordance with the review protocol. Any discrepancies between reviewers were resolved through discussion and, if necessary, consultation with a third reviewer. Studies that did not meet the inclusion criteria were excluded, and the reasons for exclusion were recorded.

Data were extracted from the included studies for assessment of study quality and evidence synthesis. Extracted information included study details (authors, year of study, country of study, research design, and type of control); participant characteristics (age, gender, sample size, and recruitment setting or method); intervention details (content, mode of delivery, number of sessions, and length of sessions); outcome measures and time points; and primary (ie, depression and anxiety symptoms) and secondary (ie, social ties) outcome data, including effect sizes, adherence and completion rates (eg, percentage of modules completed), and satisfaction scores (eg, self-report Likert scales). The secondary outcome, social ties, included measures of social support, social isolation, belonging, loneliness, social participation, social capital, and social functioning. The first author and a second reviewer extracted the data independently using a coding form, and any discrepancies identified were resolved through discussion (with a third reviewer where necessary). The pro forma coding sheet was tested for clarity before implementation, with all reviewers provided with a briefing before data extraction.

### Risk-of-Bias Assessment

In total, 2 reviewers independently assessed the risk of bias of the included studies using the Joanna Briggs Institute quality rating scale for RCTs [26]. The following two items were removed: (1) “Were participants blind to treatment assignment” and (2) “Were those delivering the treatment blind to treatment assignment.” These items were not relevant for this review as the distal nature of the interventions meant that participants were automatically unblinded to their condition.

### Effect Size Calculations

Effect sizes were calculated for depression, anxiety, and social ties at the postintervention and follow-up time points (where measured and reported). The final follow-up period reported was chosen for effect size calculations. Furthermore, where multiple measures of depression and anxiety were used, the most commonly used measures in research and practice were chosen for effect size calculations (ie, Edinburgh Postnatal Depression Scale [EPDS] and Generalized Anxiety Disorder–7). Cohen *d* [27] was calculated by 3 reviewers and cross checked with the meta-analysis output. Cohen [27] reports the conventional levels of effect sizes as small (Cohen *d*=0.2), medium (Cohen *d*=0.5), and large (Cohen *d*=0.8).

### Data Synthesis and Meta-Analysis

The included studies were initially synthesized qualitatively, with a narrative summary describing the main characteristics and results (including effect sizes). The summary focused on participant characteristics, information about the intervention, and risk of bias. On the basis of available data, studies were also quantitatively synthesized through 5 meta-analyses of intervention effects—for depression and anxiety at the postintervention and follow-up time points and for social ties only at the postintervention time point (there were insufficient studies with follow-up time points for this outcome). The meta-analyses yielded statistical summaries of the effects of the interventions at the different time points. A total of 78% (14/18) of the studies were eligible for inclusion in at least one of the meta-analyses, with varying numbers in each analysis based on whether the study measured and reported the required data. Where a journal article did not provide the required data, the authors were contacted twice over a 1-month period with a request for this information. The authors of 2 studies did not respond, and one did not provide appropriate information due to incompatible statistical methods; therefore, these studies were excluded from the meta-analyses.

RevMan (The Cochrane Collaboration) [28] was used to conduct the meta-analyses, which all used a random-effects model due to the expected high heterogeneity, allowing for the differences between the studies to be modeled [29]. In addition, the standardized mean difference (SMD) was used as multiple measurement tools were implemented to measure depression, anxiety, and social ties in the 14 studies [30]. Between-group heterogeneity was tested using the *I*^2^ statistic in RevMan. The *I*^2^ statistic and accompanying CIs indicate the level of difference between the studies by reporting heterogeneity as a percentage [31]. The levels were categorized as low (*I*^2^=25%), moderate (*I*^2^=50%), and high (*I*^2^=75%) heterogeneity [31]. Subgroup analyses were considered given the high level of heterogeneity; however, it was deemed not appropriate due to the insufficient number of studies. High heterogeneity was expected due to the many differences between the studies, including the measures used, intervention length, onset of the intervention, follow-up timing, country of origin, intervention formats, content, theoretical framework, and the fact that 11% (2/18) of the studies included couples [32]. Funnel plots were also created in RevMan to assess for publication bias, which is evident if the funnel plot is asymmetrical [33].

## Results

### Study Selection

**Figure 1:**
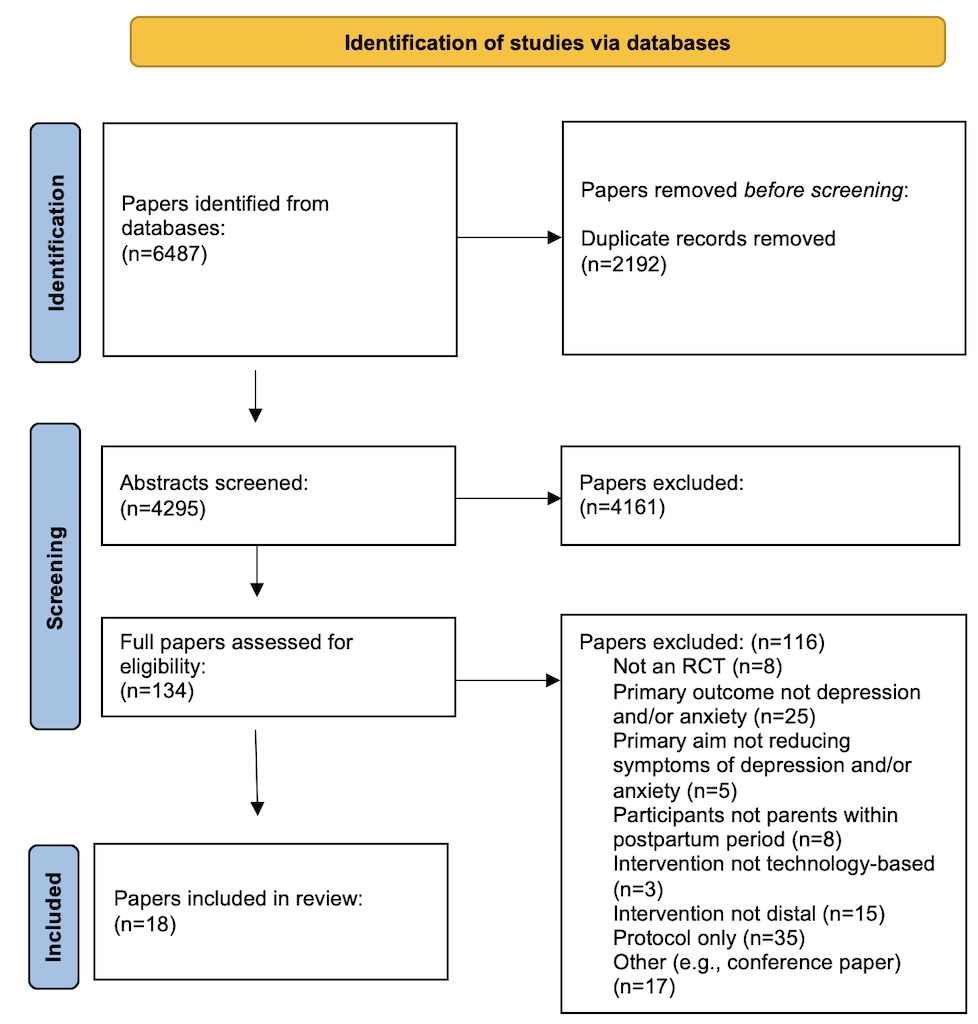
PRISMA (Preferred Reporting Items for Systematic Reviews and Meta-Analyses) flow diagram. RCT: randomized controlled trial.

Figure 1 presents the PRISMA flow diagram showing the flow of studies through the different phases of the systematic review, including identification, screening, eligibility, and inclusion. The 3 searches (original and 2 updates) identified a total of 6487 studies, of which 2192 (33.79%) were excluded as duplicates. All the remaining papers were screened and coded by 2 reviewers. A total of 4295 articles were screened by their title and abstract, with 4161 (96.88%) being deemed irrelevant and, therefore, excluded. Following screening, 134 full-text articles were collected and reviewed, of which 116 (86.6%) were excluded, and the reasons for exclusion were noted. In total, 18 papers were deemed eligible and coded for inclusion in the systematic review. Of the 18 included studies, 14 (78%) provided sufficient data for inclusion in at least one of the meta-analyses.

### Intervention Characteristics

#### Overview

Table 1 shows the intervention characteristics of the included studies. Overall, there were 18 studies deemed eligible for this review. This included studies conducted in 12 different regions between 2013 and 2023, with the largest number of studies conducted in Australia (3/18, 17%) and China (3/18, 17%).

**Table 1:** Intervention characteristics.

Intervention	Study	Region	Content	Length	Delivery mode	Host	Automated, infrequent, or optional communication
Mothers and Babies Course (Curso Mamas y Bebes)	Barrera et al [34]	Global sample	CBT^a^	8 lessons	Web	University	No
Parents Interacting with Infants	Boyd et al [35]	United States	CBT and education	8 modules over 8 weeks	Web	Not for profit	Yes: optional forum with other participants and facilitators
Be a Mom	Carona et al [36]	Portugal	CBT	5 modules over 5 weeks	Web	University	Yes: automated reminders and phone calls assessing IT and program questions and difficulties
iParent	Chan et al [37]	Hong Kong	Education	The app could be used from the first visit to the antenatal clinic until childbirth	Mobile app	Hospital	Yes: optional, unscheduled messages from a health professional answering questions related to pregnancy, childbirth, and infant health and care
MomMoodBooster2	Danaher et al [38]	United States	CBT	6 sessions over a 12-week active treatment phase; participants could continue using it for 7 months	Web	University	Yes: 1 nonclinical call to resolve any IT difficulties and 1 call after the intervention for feedback
Be a Mom	Fonseca et al [39]	Portugal	CBT	5 modules; 1 module per week recommended but could be completed at participants’ own pace	Web	University	Yes: reminders and emails for technical support
Mindful Self-Compassion on Program	Guo et al [40]	China	CBT	6-week program with 10 hours of training over 36 episodes (6 episodes per week)	Web	Hospital	No
Mamma Mia	Haga et al [41]	Norway	IPT^b^ and education	44 sessions over an 11.5-month period	Web	Hospital	No
MamaKits Online	Heller et al [42]	The Netherlands	Education and problem-solving treatment	5 modules over 5 weeks	Web	University and hospital	Yes: reminders and optional, unscheduled messages or phone calls with coaches to answer questions and receive feedback for homework tasks
Baby Steps	Kavanagh et al [43]	Australia	Education	9 modules with no advised pace	Web	University	Yes: reminders from a support person
—^c^	Lennard et al [44]	Australia and New Zealand	Compassion-focused therapy and education	Unlimited access to online resources over 8 weeks	Web	University	Yes: reminders
MUMentum Postnatal	Loughnan et al [45]	Australia	CBT	3 sessions over 6 weeks	Web	University and hospital	Yes: reminders and unscheduled messages from a health professional (only if participants scored highly on distress questionnaires as a safety protocol)
Luna Baby	Nishi et al [46]	Japan	CBT	6 sessions delivered weekly	Mobile app	University and preexisting mobile app	Yes: automated reminders
NetMums	O’Mahen et al [21]	United Kingdom	CBT and behavioral activation	11 sessions over 15 weeks	Web	University	Yes: optional online chat rooms with other participants and health professionals
CareMom	Qin et al [47]	China	CBT	28 daily challenges completed over 4 weeks	Mobile app	Hospital	Yes: reminders
Home but not Alone	Shorey et al [48]	Singapore	Education	App available over 4 weeks	Mobile app	University and hospital	No
Spirits Healing App	Sun et al [49]	China	MBCT^d^	8 sessions over 8 weeks	Mobile app	University and hospital	Yes: control group had personalized communication and intervention group had automated reminders
—	Zhang et al [50]	China	Mindfulness	6 modules delivered weekly	Mobile app	University	Yes: standardized reminders

^a^CBT: cognitive behavioral therapy.

^b^IPT: interpersonal psychotherapy.

^c^Intervention name was not provided.

^d^MBCT: mindfulness-based cognitive therapy.

#### Content

Of the 18 studies identified in the review, most (11/18, 61%) assessed a CBT-based intervention. A total of 17% (3/18) of the studies combined CBT with another therapeutic approach (eg, CBT with psychoeducation) [35] or delivered specific components of CBT (eg, behavioral activation or mindfulness-based CBT) [49,51]. One study assessed a mindfulness intervention [50]. The remaining studies (6/18, 33%) were education-focused interventions, with 50% (3/6) of these studies combining psychoeducation with non-CBT therapeutic approaches, specifically IPT [41], problem-solving therapy [42], and compassion-focused therapy [44].

#### Format and Mode of Delivery

All the interventions (18/18, 100%) were delivered to the individual (vs being group based), and most of the studies (12/18, 67%) tested online interventions that were directly accessed or downloaded from the internet. The remaining studies (6/18, 33%) evaluated interventions that were delivered as mobile apps.

#### Automated, Infrequent, or Optional Communication

Most of the interventions (14/18, 78%) included optional communication with the participants. A significant proportion of this communication was reminders to complete the program (10/18, 56%). A total of 11% (2/18) of the trials [35,51] included optional online forums with other participants and health professionals or facilitators. In addition, 11% (2/18) of the studies [37,42] had messages from health professionals answering questions related to pregnancy and birth. One study (1/18, 6%) [45] allowed for communication with a health professional if the participant expressed high levels of distress in their questionnaire as a safety precaution. In total, 11% (2/18) of the studies [36,38] provided nonclinical phone calls to seek feedback on any technological issues and the participants’ experience with the program. The remaining studies (4/18, 22%) provided no communication as part of the intervention or trial.

#### Intervention Host

Most of the interventions (13/18, 72%) were offered by a university. Of those 13 studies, 4 (31%) were cohosted by a hospital, and 1 (8%) was cohosted by a preexisting mobile app. Of the remaining 5 interventions, 4 (80%) were hosted by hospitals, and 1 (20%) was hosted by a not-for-profit child health organization.

#### Length of the Intervention

The length of the interventions included in this review was reported in terms of duration or number of intervention modules. Of those that reported the number of modules (15/18, 83%), 8 modules (3/18, 17%), 6 modules (3/18, 17%), and 5 modules (3/18, 17%) were the most common. The remaining 40% (6/15) of the interventions had 3 to 44 modules. Regarding the time allowed to complete the modules, 11% (2/18) of the studies did not specify a suggested time frame, 39% (7/18) of the studies recommended 1 module per week for a set duration, 11% (2/18) of the studies recommended multiple modules per week for a set duration, 22% (4/18) of the studies recommended 1 module over multiple weeks, and 17% (3/18) of the studies provided unlimited access over a set number of weeks. The overall intervention duration ranged from 4 to 11.5 weeks, with 6 weeks being the most common (4/18, 22%) followed by 8 weeks (3/18, 17%) and 5 weeks (3/18, 17%).

### Trial Characteristics and Outcomes

#### Overview

Table 2 shows the trial characteristics and outcomes. Overall, the interventions included 11,802 participants. Of those, 5916 were part of the intervention conditions, and 5886 were part of the control conditions. The sample sizes of each study ranged from 24 to 5017, with a median sample size of 221.

**Table 2:** Trial characteristics and outcomes.

Study	Recruitment setting	Control group	Gender	Participant (parent) age (y) and child age (months), mean (SD)	Target population	Sample size	Outcomes	Quality rating score
Barrera et al [34]	Online advertising	Attention	100% female	Parent: 30.19 (5.57); child: not reported	Universal: birth mothers of first or subsequent children	Intervention: 57; control: 54	Depression: small effect (Cohen d=–0.04) at the postintervention time point and medium effect size (Cohen d=–0.54) at the 6-month follow-up. No significant effect (*P*=.11). Satisfaction: 28/57 of the participants rated the lesson material. In total, 88.9% indicated that the lessons were helpful for managing mood changes, whereas the content was rated as easy to understand (mean 4.12, SD 1.02) and highly useful (mean 4.20, SD 1.10).	8/11
Boyd et al [35]	Pediatric primary care clinic	Attention	100% female	Parent: 26.4 (1.9); child: 2.7 (0.2)	Indicated: birth mothers	Intervention: 12; control: 12	Depression: large significant effect (Cohen d=–0.82) at the postintervention time point (*P*<.01). Adherence: all the mothers (100%) in the social media group attended at least one session. Average attendance was 83%, and average participant commenting was 73%. Satisfaction: the mean ratings of individual sessions ranged from 3.6 to 4.4 out of 5, demonstrating favorable scores. The mean satisfaction score for the overall intervention was 4.54 (SD 0.78; 91%).	6/11
Carona et al [36]	Online advertising	Waitlist and TAU^a^	100% female	Parent: 32.71 (4.53); child: 2.03 (0.96)	Selective: birth mothers of first or subsequent children with or without a history of mental illness	Intervention: 542; control: 511	Depression: medium significant effect (Cohen d=–0.42) at the postintervention time point (*P*<.001). Anxiety: medium significant effect (Cohen d=–0.41) at the postintervention time point (*P*<.001).	9/11
Chan et al [37]	Hospital	TAU	100% female	Parent: 31.25 (4.55); child: not reported	Indicated and selective: birth mothers of first children with or without a history of mental illness	Intervention: 330; control: 330	Depression: small significant effect (Cohen d=–0.13) at the postintervention time point (*P*=.049). Anxiety: small nonsignificant effect (Cohen d=0.05) at the postintervention time point (*P*=.94).	10/11
Danaher et al [38]	Prenatal clinic and hospital	TAU	100% female	Parent: 31.9 (5.3); child: not reported	Indicated: birth mothers of first and subsequent children with a history of mental illness	Intervention: 96; control:95	Depression: small significant effect (Cohen d=–0.33) at the postintervention time point (*P*=.003). Anxiety: medium nonsignificant effect (Cohen d=–0.39) at the postintervention time point (*P*=.28). Adherence: mean 10.3 (SD 8.7) total program visits; mean 4.3 (SD 2) session visits; 49% viewed all 6 sessions Satisfaction: 96% rated the program as somewhat to extremely easy to use; 83% rated it as somewhat to extremely helpful; 93% would recommend the program	10/11
Fonseca et al [39]	Hospital and online advertising	Waitlist	100% female	Parent: 32.58 (4.82); child: 2 (0.89)	Universal: birth mothers of first or subsequent children with or without a history of mental illness	Intervention: 98; control: 96	Depression: small nonsignificant effect size (Cohen d=0.01) at the postintervention time point (*P*=.61). Anxiety: small nonsignificant effect (Cohen d=–0.08) at the postintervention time point (*P*=.90). Social ties: small nonsignificant effect (Cohen d=–0.14) at the postintervention time point (*P*=.51). Adherence: 41.8% of the participants completed the program. Satisfaction: compared to noncompleters, completers were significantly more satisfied (*P*<.001), had a higher intention to use the intervention if needed (*P*<.001), and perceived the program as useful (*P*=.006). No significant difference was reported in the perceived demandingness of using the program (*P*=.62).	8/11
Guo et al [40]	Prenatal clinic	Waitlist	100% female	Parent: 30.6 (5.95); child: not reported	Indicated: birth mothers	Intervention: 157; control: 157	Depression: significant effect at the 3-month and 1-year follow-ups (*P*<.01). Anxiety: not reported. Adherence: the overall attendance rate was 91.8%, with no significant difference between the groups (*P*=.56). Satisfaction: 95% of the participants filled out the posttest survey, the results of which indicated high acceptability.	9/11
Haga et al [41]	Prenatal clinic and hospital	TAU	100% female	Parent: 31 (4.6); child: not reported	Universal: birth mothers of first or subsequent children; mothers with or without a history of mental illness	Intervention: 678; control: 664	Depression: small effect size (Cohen d=–0.10) at the postintervention time point. The intervention group showed less severe depression symptoms than participants in the control group on all measurement occasions after baseline. This difference was statistically significant at gestational week 37 (*P*=.008) and 6 weeks post partum (*P*=.03). Adherence: 33% of the participants in the intervention group completed all 44 sessions, 51% completed ≥36 sessions, and 6% completed no sessions.	9/11
Heller et al [42]	Community advertising, prenatal clinic, online advertising, and obstetricians and midwives	TAU	100% female	Parent: 32.08 (4.61); child: not reported	Indicated: birth mothers of first or subsequent children; mothers with or without a history of mental illness	Intervention: 79; control: 80	Depression: small nonsignificant effect sizes reported at the postintervention time point and 6-week postpartum follow-up using the CES-Db (postintervention time point: Cohen d=0.09; 6-week postpartum follow-up: Cohen d=–0.27) and EPDSc (postintervention time point: Cohen d=0.11; 6-week postpartum follow-up: Cohen d=–0.12). Anxiety: small nonsignificant effect sizes were reported at the postintervention time point (Cohen d=–0.05) and 6-week postpartum follow-up (Cohen d=–0.18). Adherence: 47% completed all modules. Satisfaction: 87% were satisfied with the help they received, and 74% would recommend the intervention to others.	10/11
Kavanagh et al [43]	Community advertising, prenatal clinic, hospital, and referral	Attention	50% female and 50% male	Parent: 32.2 (4.4); child: not reported	Universal: birth mothers and fathers (coparenting couples) of first children with or without a history of mental illness	Intervention: 124; control: 124	Depression: small nonsignificant effect sizes were reported at the postintervention time point for mothers (Cohen d=0.01) and fathers (Cohen d=0.01) and at the 6-month follow-up for mothers (Cohen d=0.14) and fathers (Cohen d=0.14). Social ties: small nonsignificant effect sizes were reported at the postintervention time point for mothers (Cohen d=0.10) and fathers (Cohen d=0.10) and at the 6-month follow-up for mothers (Cohen d=0.14) and fathers (Cohen d=0.17). Adherence: 37.3% of participants accessed their programs more than once, with higher rates for mothers (53.6%) than fathers (20.9%). Satisfaction: satisfaction with the program was high among the participants who accessed it at least once (median satisfaction score 75/100; 92% satisfaction score ≥50/100), with no significant differences due to treatment or parent gender.	9/11
Lennard et al [44]	Online advertising and forums	Waitlist	100% female	Parent: 32.56 (3.96); child: 9.54 (6.59)	Universal: birth mothers of first or subsequent children with or without a history of mental illness	Intervention: 231; control: 239	Depression: small nonsignificant effect size (Cohen d=–0.21) at the postintervention time point (*P*=.11). Anxiety: small nonsignificant effect size (Cohen d=0.02) at the postintervention time point (*P*=.45). Adherence: 64.9% of the intervention group reported watching the psychoeducational video at least once (18.7% more than once) and did the guided self-compassion exercise (13.2% more than once). Satisfaction: overall, 69.3% reported attempting to apply the strategies in their own lives. A total of 47.3% reported feeling that they had become more self‐compassionate over the study period. Most agreed that self‐compassion would be helpful for new mothers coping with challenging birth (90.1%) and breastfeeding (83.5%) experiences. Similarly, 75.8% of women reported that the SMS text message reminders were useful. Finally, 80.2% would recommend this type of intervention to others.	7/11
Loughnan et al [45]	Community advertising, prenatal clinic, online advertising, and online forum	TAU	100% female	Parent: 32.56 (4.53); child: 4.55 (3.05)	Indicated: birth mothers of first or subsequent children with or without a history of mental illness	Intervention: 69; control: 62	Depression: large significant effect sizes using the PHQ-9d (Cohen d=–1) and EPDS (Cohen d=–0.91) at the postintervention time point (*P*<.05). Large significant effect sizes using the PHQ-9 (Cohen d=–0.86) and EPDS (Cohen d=–0.99) at the 4-week follow-up (*P*<.05). Anxiety: large significant effect size (Cohen d=–0.78) at the postintervention time point (*P*<.05). A large significant effect size (Cohen d=–1.15) at the 4-week follow-up (*P*<.05). Adherence: a total of 46 women completed all 3 lessons of treatment (46/61, 75% completion rate). Of those in iCBTe, 82% completed posttreatment questionnaires, and 61% completed follow-up questionnaires. Of those in TAU, 85% and 76% provided posttreatment and follow-up data, respectively. Satisfaction: 86% judged the quality of the program as good to excellent. A total of 80% reported being mostly to very satisfied with the program	9/11
Nishi et al [46]	Message sent through preexisting mobile app	No intervention	100% female	Parent: 30.44 (4.6); child: not reported	Universal: birth mothers of first and subsequent children with or without a history of mental illness	Intervention: 2509; control: 2508	Depression: 0 cases had an MDEf at baseline, and 59 cases (2.35%) in the intervention group and 73 cases (2.91%) in the control group experienced the onset of an MDE during the intervention and 3-month postpartum follow-up period. Adherence: 37.2% completed all 6 modules. Module completion rates ranged from 55.9% to 79.5%.	9/11
O’Mahen et al [21]	Online advertising	TAU	100% female	Parent: 32.3 (4.7); child: not reported	Indicated: birth mothers of first or subsequent children with or without a history of mental illness	Intervention: 462; control: 448	Depression: medium significant effect (Cohen d=–0.55) at the postintervention time point (*P*<.01). Adherence: number of sessions viewed ranged between 95 and 1310, with a decrease each session except for session 7. Engagement with the treatment chat room and online clinic was low. A total of 7% (32/462) of the women posted on the chat room. Satisfaction: key acceptability endorsements were regarding the flexible and convenient delivery of the treatment and helping women help themselves, although the women also noted that they struggled to keep up with the program.	8/11
Qin et al [47]	Hospital	Waitlist and TAU	100% female	Parent: 31.9 (3.62); child: not reported	Healthy population: birth mothers of first and subsequent children with no history of mental illness	Intervention: 57; control:55	Depression: medium significant effect (Cohen d=–0.55) at the postintervention time point (*P*=.04). Anxiety: small nonsignificant effect (Cohen d=–0.29) at the postintervention time point (*P*=.19). Adherence: 90% completed all 28 daily challenges. Satisfaction: overall satisfaction—mean 4.58/5 (SD 0.74); recommendation to a friend—mean 4.54/5 (SD 0.80); relatedness to life—mean 4.44/4 (SD 0.62); application of content to life—mean 4.44/5 (SD 0.58)	5/11
Shorey et al [48]	Hospital	TAU	50% female and 50% male	Parent: 32.66 (5.03); child: not reported	Universal: birth mothers and fathers (coparenting couples) of first or subsequent children with or without a history of mental illness	Intervention: 126; control: 124	Depression: no significant effect. Social ties: the intervention group had statistically significant improvements for social support at 4 weeks post partum compared with the control group. This occurred regarding support from spouses (*P*<.001) and other sources (*P*<.001). Satisfaction: most of the participants in the intervention group felt satisfied with the mHealthg app intervention (n=97, 77%). Most of the participants in the intervention group also stated that they benefited from the mHealth app intervention (n=94, 74.6%)	9/11
Sun et al [49]	Hospital	Attention	100% female	Parent: 29.91 (4.02); child: not reported	Indicated: birth mothers of first or subsequent children with or without a history of mental illness	Intervention: 84; control: 84	Depression: a medium nonsignificant effect size was reported (Cohen d=–0.48) at the postintervention time point (*P*=.25), and a small nonsignificant effect size was reported (Cohen d=0.11) at the 6-week postpartum follow-up. Anxiety: small nonsignificant effect sizes (Cohen d=–0.27) at the postintervention time point (*P*=.75) and at the 6-week postpartum follow-up (Cohen d=–0.08). Adherence: 8% completed the intervention.	9/11
Zhang et al [50]	Prenatal clinic	TAU	100% female	Parent: 30.29 (4.29); child: 0-6 months	Indicated: birth mothers of first and subsequent children with a history of mental illness	Intervention: 80; control: 80	Depression: medium significant effect (Cohen d=–0.55) at the postintervention time point (*P*<.001) and final 6-month postpartum follow-up (Cohen d=–0.48; *P*<.001). Significant effects reported at follow-ups—gestational weeks 36-37 (*P*<.001), 6 weeks post partum (*P*<.001), and 3 months post partum (*P*=.001). Anxiety: large significant effect (Cohen d=–0.85) at the postintervention time point (*P*<.001) and a medium effect (Cohen d=–0.53) at the final 6-month postpartum follow-up (*P*=.03). Significant effects reported at gestational weeks 36-37 (*P*<.001), 6 weeks post partum (*P*<.001), and 3 months post partum (*P*=.02).	10/11

^a^TAU: treatment as usual.

^b^CES-D: Center for Epidemiologic Studies Depression Scale.

^c^EPDS: Edinburgh Postnatal Depression Scale.

^d^PHQ-9: Patient Health Questionnaire–9.

^e^iCBT: internet-based cognitive behavioral therapy.

^f^MDE: major depressive episode.

^g^mHealth: mobile health.

#### Recruitment Setting and Clinical Interview

Most of the studies (11/18, 61%) used 1 recruitment setting, whereas the remaining studies (7/18, 39%) used a combination of recruitment settings. Across the studies, participants were recruited through hospitals (8/18, 44%), online advertising (7/18, 39%), online forums (2/18, 11%), prenatal clinics (7/18, 39%), community advertising (3/18, 17%), and messaging via an existing mobile app (1/18, 6%). One study included a clinical interview to determine eligibility in the form of a web-based and self-administered World Health Organization Composite International Diagnostic Interview [46].

#### Participant Age and Gender and Target Population

The target populations varied across the studies. Indicated interventions (ie, delivered to individuals with elevated symptoms) were the most common (8/18, 44%), followed by universal interventions (7/18, 39%), selective interventions (1/18, 6%), combined indicated and selective prevention interventions (1/18, 6%), and interventions targeted at a healthy population (1/18, 6%). The vast majority of studies (16/18, 89%) included mothers only, with only 11% (2/18) of the studies including coparenting mothers and fathers. All 18 studies were targeted at birth parents only, with most (14/18, 78%) including both first-time parents and those with ≥2 children. A small proportion of studies (2/18, 11%) targeted first-time parents only. A total of 33% (6/18) of the studies reported on the age of the children at the time of the intervention, with the mean age ranging from 0 to 9.54 months. Of these 6 studies, 3 (50%) included children with a mean age of 2 months.

Regarding parental mental health history, most studies (13/18, 72%) indicated that their participants were parents both with a history of mental illness and without. A total of 11% (2/18) of the studies targeted parents with a history of mental illness, and 6% (1/18) of the studies enrolled only participants with no history of mental illness. The remaining studies (2/18, 11%) did not report on these factors. Mean parent age ranged between 26.4 and 39.9 years across the studies.

#### Control Group and Randomization

Of the 18 studies included, almost half (n=10, 56%) had a “treatment as usual” control group, making it the most common type. Treatment as usual tended to include information about pregnancy and childbirth alongside regular appointments with health professionals. The second most common type of control group was “attention” control groups (4/18, 22%), which involved receiving information about the postpartum period, and waitlist control groups (5/18, 28%). One study included a “no intervention” control, which differed from the waitlist and treatment-as-usual controls by providing no treatment, including after the study. A total of 89% (16/18) of the studies used individual randomization, and the remaining 11% (2/18) of the studies, which included couples, used stratified randomization. Participants completed the interventions individually, including couples, although the latter could discuss the intervention.

#### Measurement Time Point

The measurement time points varied between the studies. Exclusively pre- and postintervention measures were evident in 44% (8/18) of the studies. The remaining 56% (10/18) of the studies included pre- and postintervention measures in addition to measures at other time points, ranging from 4 weeks to 6 months after the intervention. The studies included preintervention measurements during pregnancy (11/18, 61%) and post partum (7/18, 39%) depending on the timing of the intervention regarding antenatal and postnatal onset.

#### Outcomes

##### Depression

Depression was measured in all the studies (18/18, 100%), with the most frequently used scale being the EPDS (14/18, 78%). A total of 29% (4/14) of these studies used additional depression measures, including the Beck Depression Inventory (1/4, 25%), Patient Health Questionnaire–9 (1/4, 25%), and Center for Epidemiologic Studies Depression Scale (2/4, 50%). Another 29% (4/14) of these studies used the Beck Depression Inventory–II (1/4, 25%); Depression, Anxiety, and Stress Scales–21 (DASS-21; 1/4, 25%); Patient Health Questionnaire–9 (1/4, 25%); and World Health Organization Composite International Diagnostic Interview (1/4, 25%).

All 18 studies reported a decrease in symptoms in the intervention group at the postintervention time point. This decrease was significant in 50% (9/18) of the studies when compared to the control groups, with their effect sizes ranging from Cohen *d*=–0.10 [41] to Cohen *d*=–0.91 [45] (median –0.55). Among these 9 studies, multiple control group types were used, including attention (n=1, 11%), treatment as usual (n=6, 67%), and a combination of waitlist and treatment as usual (n=2, 22%). A total of 50% (9/18) of the studies reported a follow-up period of between 4 weeks and 6 months, with all indicating a decrease in symptoms of depression in the intervention group. Of these 9 studies, 4 (44%) reported a significant difference compared to the control group, with effect sizes ranging from Cohen *d*=–0.10 [41] at the 6-month follow-up to Cohen *d*=–0.99 [45] at the 4-week follow-up (median –0.48).

##### Anxiety

Anxiety was measured in 61% (11/18) of the studies. The most commonly used anxiety scales were the Generalized Anxiety Disorder–7 (4/11, 36%), DASS-21 (3/11, 27%), and Hospital Anxiety and Depression Scale–A (3/11, 27%). One study used the State-Trait Anxiety Inventory (1/11, 9%).

All studies reported a reduction in symptoms of anxiety in the intervention groups at the postintervention time point, with 27% (3/11) of the studies [36,45,50] reporting significant effects with moderate to large effect sizes relative to the control group (Cohen *d*=–0.41, Cohen *d*=–0.78, and Cohen *d*=–0.85). Of these 3 studies, all (100%) used a treatment-as-usual control condition, and 1 (33%) additionally included a waitlist control condition. In total, 4 studies reported a follow-up period of between 4 weeks and 6 months, of which 2 (50%) [45,50] reported a significant effect (Cohen *d*=–1.15 at the 4-week follow-up; Cohen *d*=–0.53 at the 6-month follow-up).

##### Social Ties

Social ties were measured in 17% (3/18) of the studies. Each study used a different measure, which included the Portuguese version of the Revised Dyadic Adjustment Scale, short Medical Outcomes Study–Social Support Survey, and the Perceived Social Support for Parenting Scale.

Of these studies, only the one by Shorey et al [48] reported a significant increase in social support at the postintervention time point; however, it did not provide sufficient data to calculate the size of the effect. In addition, only the study by Kavanagh et al [43] assessed social ties at the 6-month follow-up, noting a nonsignificant effect, resulting in insufficient studies to complete a meta-analysis for this time point.

##### Adherence

Adherence was measured in 72% (13/18) of the studies using differing methods. Multiple studies (9/18, 50%) assessed adherence using data collected automatically by the intervention program, including number of modules completed, pages accessed, log-ins, time between log-ins, time spent on the app, and completion rate. A total of 17% (3/18) of the studies administered a self-report questionnaire after the intervention, which included various questions about the use of the app and how they used what they learnt in their lives. One study included a viewing session on social media and the chance to post comments and used this interaction as an adherence measure. Among the 13 studies measuring adherence, attendance and program access ranged from 7% [21] to 91.8% [40] (n=5, 38% of the studies; median 64.9%). Completion of the intervention ranged from 8% [49] to 90% [47] of the participants (8/13, 62% of the studies; median 44.4%).

##### Satisfaction

Satisfaction was measured in 72% (13/18) of the studies. The most common method of measurement (6/13, 46% of the studies) was administering postintervention scales created by the researchers, including questions about how useful and understandable the program material was, how likely the participants were to use it in the future, and their satisfaction with the intervention. Individualized postintervention self-report questionnaires were also commonly used (4/13, 31%). In total, 2 validated questionnaires used by 3 studies were the Client Satisfaction Questionnaire (n=1, 33%) and Telemedicine Satisfaction Questionnaire (n=1, 33%). The remaining study used completion rate as an indicator of the acceptability of the intervention.

Among the 6 studies who measured it, participant satisfaction ranged from 77% [48] to 92% [47] (n=5, 83% of the studies; median 87%), whereas 74% [42] to 93% [38] of the participants (n=4, 67% of the studies; median 85.6%) reported that they would recommend the intervention to others. Reports of benefitting from the program and finding it helpful ranged between 74.6% and 88.9% (3/13, 23% of the studies; median 83%). Fonseca et al [39] noted that those who completed the intervention were more satisfied with the program, had a higher intention to use the program in the future if needed, and perceived the intervention as useful at a significantly higher level than those allocated to the intervention condition who did not complete the program.

### Quality Rating Score

The quality rating scores are presented in Table 2 and ranged between 6 and 10 out of 11. The most frequent score was 9 (8/18, 44% of the studies; median 9), indicating generally high methodological quality. The criteria most commonly met were true randomization, outcomes measured in the same way across groups, and outcomes measured reliably (18/18, 100%), and the criteria least commonly met were follow-up complete and differences between groups at follow-up described and analyzed (11/18, 61%). Multimedia Appendix 3 [21,34-50] provides detailed quality rating scores.

### Synthesis of Results

#### Primary Analysis

**Figure 2:**
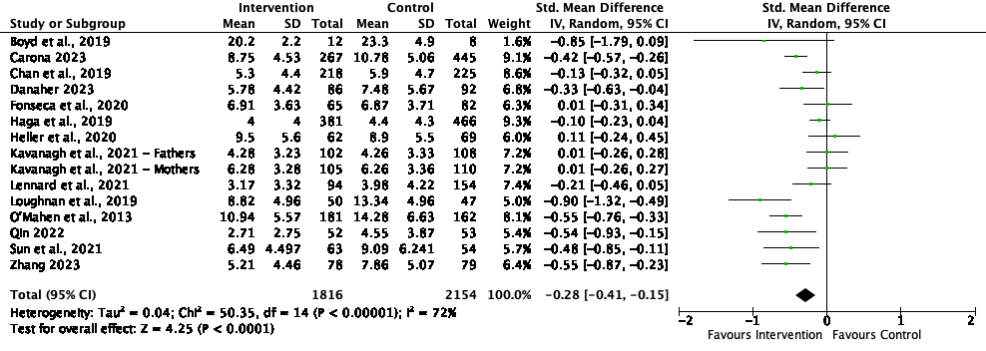
Depression meta-analysis and forest plot at the postintervention time point. IV: inverse variance. [21,35-39,41-45,47,49-50].

**Figure 3:**
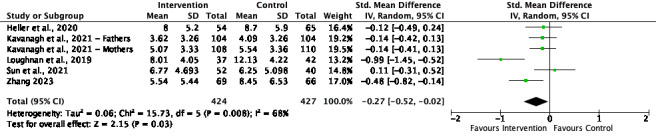
Depression meta-analysis and forest plot at follow-up. IV: inverse variance. [42-43,45,49-50].

**Figure 4:**
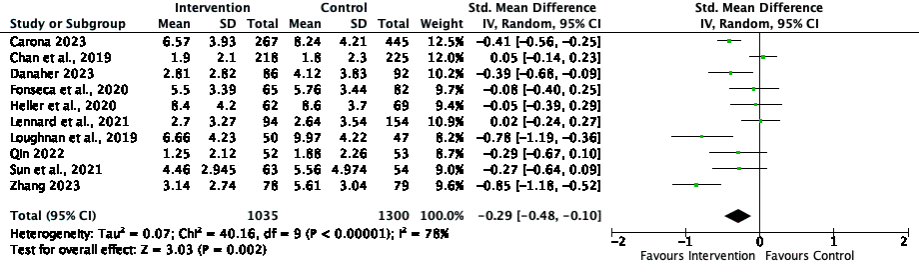
Anxiety meta-analysis and forest plot at the postintervention time point. IV: inverse variance. [36-39, 42, 44-45, 47, 49-50].

**Figure 5:**
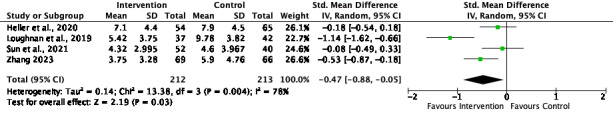
Anxiety meta-analysis and forest plot at follow-up. IV: inverse variance. [42, 45, 49-50].

**Figure 6:**
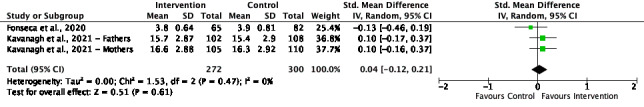
Social tie meta-analysis and forest plot at the postintervention time point. IV: inverse variance. [39, 43].

Figures 2-6 present the forest plots for the 5 meta-analyses conducted to assess the effect of the interventions on depression, anxiety, and social ties.

#### Depression

The overall effect size for depression at the postintervention time point was small and significant (SMD=–0.28, 95% CI –0.41 to –0.15; *P*<.001), with high and significant heterogeneity (*I*^2^=72%; *P*<.001). The effect size at follow-up was small and significant (SMD=–0.27, 95% CI –0.52 to –0.02; *P*=.03), with high and significant heterogeneity (*I*^2^=68%; *P*=.008).

#### Anxiety

The effect size for anxiety at the postintervention time point was small and significant (SMD=–0.29, 95% CI –0.48 to –0.10; *P*=.002), with high and significant heterogeneity (*I*^2^=78%; *P*<.001). The effect size at follow-up was medium and significant (SMD=–0.47, 95% CI –0.88 to –0.05; *P*=.03), with high and significant heterogeneity (*I*^2^=78%; *P*=.004).

#### Social Ties

The effect size for social ties at the postintervention time point was small and nonsignificant (SMD=0.04, 95% CI –0.12 to 0.21; *P*=.61), with low and nonsignificant heterogeneity (*I*^2^=0%; *P*=.47). Due to a lack of studies, a meta-analysis for the follow-up time point could not be conducted.

#### Publication Bias

Publication bias was analyzed using funnel plots for each meta-analysis (Multimedia Appendices 4-8). There appeared to be symmetry in these funnel plots, indicating minimal publication bias.

## Discussion

### Principal Findings and Comparison to Prior Work

This review and series of meta-analyses aimed to assess the effectiveness of technology-based distal interventions in reducing or preventing symptoms of postpartum depression or anxiety and increasing social ties and to determine the level of adherence and satisfaction associated with these interventions. Overall, 18 relevant studies were identified, 14 (78%) of which were able to be included in one or more of the meta-analyses. The overall results of the meta-analyses were promising, with significant effects in favor of the intervention condition found for depression and anxiety at the postintervention and follow-up time points. These findings align with the outcomes of previous systematic reviews and meta-analyses of digital mental health interventions for perinatal depression [22-24] and anxiety in women [23,24], which also found small to medium overall effects in favor of the intervention condition. This review extends the findings of previous reviews through its assessment of interventions for both mothers and fathers and by expanding the focus beyond CBT-based interventions.

Most of the studies that showed efficacy in this review compared the focal intervention to an attention or treatment-as-usual control condition. Overall, the findings suggest that distally delivered technology-based interventions can be effective in reducing symptoms of depression and anxiety primarily among birth mothers and provide support for their ongoing development and implementation as an alternative treatment and prevention approach to face-to-face services, which report broadly similar findings [52,53]. Given the small number of trials that used attention or active control conditions, future studies should consider using such controls to further strengthen the evidence base.

No significant effect was observed in the meta-analyses for social ties at the postintervention time point, although the findings were in the expected direction in favor of the intervention condition. Fewer studies assessed social ties in comparison to depression and anxiety, which may have contributed to the nonsignificant effects on this outcome. Previous research has shown that strong social ties tend to have a positive impact on postpartum mental health [54,55]. In addition, social isolation has been reported as a contributing factor to developing and maintaining depression and anxiety symptoms [10]. Despite this, few papers that were eligible for this review measured social ties. This resulted in not enough research to draw strong conclusions; however, it was indicated that couples participating in interventions at the same time potentially allows for increased social support [43]. This may be a beneficial area for future research to investigate to determine whether coparenting mothers and fathers’ postpartum mental health could be improved by couples completing interventions at the same time and then discussing the materials. However, it may also be the case that other forms of social or group interventions are more efficient in promoting social ties among new parents [56].

Two studies, those by Loughnan et al [45] and Boyd et al [35], produced large and significant effects for reducing symptoms of depression. These studies were both conducted with birth mothers in Western countries using online CBT programs compared to treatment-as-usual or attention control conditions. These interventions also included a form of optional communication, including messages reminding participants to complete the program, messages from health professionals if participant distress was noted as high, and optional access to a forum with other participants and facilitators. In addition, they were both short-term interventions spanning between 6 and 8 weeks. The large and significant effects demonstrated by these 2 studies suggest that these particular interventions may include effective characteristics for reducing postpartum depression or anxiety that warrant further investigation. A key area may be determining whether a limited amount of optional communication, as optimized in the interventions by Loughnan et al [45] and Boyd et al [35], is more beneficial for reducing symptoms of depression than no communication. Given this finding, it is recommended that future research directly compare the benefits of communication by having 2 intervention conditions, with one receiving communication and the other not.

The study by Zhang et al [50] produced a large and significant effect size for reducing anxiety symptoms. This study was conducted in China and focused on mindfulness via a mobile app. It included 6 modules delivered weekly and provided standardized reminder messages. This large and significant effect size indicates that the features of this intervention may be effective in reducing symptoms of anxiety using distal and technology-based interventions. As with the studies by Loughnan et al [45] and Boyd et al [35], the intervention was short term, included communication via a reminder message, and was tested among birth mothers. However, there were some differences among the 3 studies, including the fact that one was a mindfulness-based mobile app in China being used for anxiety and the other 2 were online CBT interventions in Western countries being used for depression. Future research may benefit from testing the interventions’ effectiveness on wider populations, including birth fathers and adoptive parents, which may assist in understanding whether these intervention characteristics are effective for populations other than birth mothers.

The overall methodological quality of the studies was high, indicating that measures were in place to avoid potential bias, including true randomization methods and measuring outcomes reliably, and that study authors adequately reported on their study methods. Some areas that could be improved include ensuring that the groups were similar at baseline and including follow-up measures in the study design.

Despite the scope of this review including interventions for postpartum depression and anxiety for birth and adoptive mothers and fathers, the literature search did not find any relevant papers for adoptive parents or fathers and found only 2 studies that assessed interventions targeted at both parents in coupled families. This finding suggests that there is a clear gap in the provision of interventions to support adoptive families and birth fathers who may be experiencing postpartum anxiety and depression. There is a need to develop and evaluate such interventions given the rates of disorder in these populations [6,7]. Excluding these populations from intervention research may increase their risk of poor mental health outcomes, including sleep disturbance, the breakdown of relationships, and challenges bonding with their children, in addition to developing postpartum depression and anxiety [11,12,14].

### Limitations

The results of this review should be interpreted in light of the limitations of both this paper and the included studies. First, there was high and significant heterogeneity in the 4 depression and anxiety meta-analyses. This was expected due to the many differences between the studies, including the measures used, intervention length, onset of intervention, participants, use of prevention and treatment interventions, adherence, follow-up timing, country where the studies were conducted, intervention formats, content, and theoretical perspectives. Given this, subgroup and moderator analyses were considered; however, they were deemed inappropriate due to having an insufficient number of studies. Some heterogeneity is unavoidable due to differences in populations and intervention characteristics. However, heterogeneity in measurement of anxiety, depression, social ties, adherence, and satisfaction may be addressed through further evaluation of measurement properties, harmonization of measures [57], and consideration of the limitations of some common measures [58].

Second, most studies had different follow-up measurement time points. Therefore, the statistics used for the follow-up meta-analyses were collected at different times after the intervention. This may result in some interventions appearing more or less effective than others based on the timing of the measures. Future research may benefit from determining the optimal time to measure follow-ups (eg, timing based on when participants gave birth or after the intervention has been administered or drawing on theory and evidence on the timing for the development of depression and anxiety during this period to inform these decisions), completing subgroup analyses based on follow-up time points for future meta-analyses, or conducting individual patient data meta-analyses that can accommodate variable timing in outcome measures.

A limitation of the studies included is that many of them were underpowered to detect modest effects. With universal prevention interventions, it is unlikely that medium or large effects will be identified as a large proportion of the sample will not have scope for a reduction in symptoms. Therefore, future research would benefit from the use of relatively large samples. Larger samples may also enable more nuanced examination of which groups of postpartum parents benefit most from internet-based interventions and when the interventions should be optimally delivered.

The EPDS was the most commonly used measure of depression in the included studies. This is unsurprising given that it has been largely used in previous research and postpartum support services. However, it has been criticized for its inclusion of ambiguous items, exclusion of particular forms of distress, challenges with scoring, poor predictive ability, and limited detection of depressive and anxious symptoms in men [58]. Future research may consider including either multiple or multidimensional measures (eg, the DASS-21) so that findings related to both anxiety and depression can be tested and triangulated.

Some relevant studies may not have been identified due to only including papers written in English and published in peer-reviewed journals. In addition, due to manually screening papers, relevant studies may have been excluded. However, double screening and coding protocols were in place to minimize the risk of this occurring. Further, incomplete data prevented the inclusion in the meta-analyses of several of the studies included in this review. This resulted in relevant papers not having their interventions assessed for effectiveness on symptom reduction. Although the corresponding authors were contacted with requests for data, they either did not respond or provided data that were incompatible with the meta-analysis due to the statistical analysis methods used.

### Practical Implications

There are multiple key practical implications from this review. Primarily, the results indicate promising findings to prevent and reduce postpartum anxiety and depression using distal technology-based interventions, which can be more readily accessed and widely disseminated to those in need. The provision of such interventions could allow more parents to access support when it is most needed, with reductions in anxiety and depression leading to a range of positive effects on parents and their children [20]. These interventions can also overcome the stigma often associated with mental health problems and help seeking. As such, there is a clear need to promote these interventions within health care settings and among new parents to ensure access to and awareness of them. Future research may endeavor to compare the effect of distal technology-based interventions with in-person therapies rather than control groups to further determine the comparable effectiveness of these intervention methods.

In addition, this review did not identify any distal technology-based approaches other than online interventions or mobile apps, such as podcasts or artificial intelligence–based interventions [59]. Emerging technologies may build on the benefits of web-based and mobile app interventions by additionally providing an option for those with low literacy or potentially tailoring content to individual needs and preferences. The use of audio interventions (eg, podcasts) may also increase adherence as parents can listen to the content while completing other daily activities, such as feeding their child, driving, or exercising. This may be a beneficial direction for future research to explore to allow for additional flexibility in interventions for postpartum anxiety and depression.

Finally, this review provided insights regarding the content of distal technology-based interventions. Most of the interventions were CBT based, with multiple incorporating mindfulness, and these included the 3 most effective interventions in reducing symptoms of depression and anxiety when compared to the control groups [35,45,50]. This indicates that CBT-based and mindfulness interventions can be beneficial for parents in the postpartum period, which aligns with research on nondistal or non–technology-based interventions [60,61]. In contrast, only 1 study used IPT despite this being a well-accepted therapeutic technique for this population in nondistal or non–technology-based interventions [62,63]. Future research would benefit from further assessing IPT-based distal technology-based interventions.

### Conclusions

Overall, the findings of this review and the meta-analyses suggest that distal technology-based interventions are effective in reducing symptoms of postpartum anxiety and depression when compared to a control condition for birth parents, particularly mothers. Although the overall effect sizes were small to medium, there was a consistent reduction in symptoms in the intervention groups. Short-term interventions with optional communication channels, such as reminders to complete the program, were associated with the largest effects. Further research is required to determine what factors make some interventions more effective than others and apply this to adoptive parents, birth fathers, and couples in addition to birth mothers. Furthermore, while there is a growing body of research investigating distal interventions for postpartum depression and anxiety, there has been limited focus on social ties. This is an area for future research given the prevalence of social isolation in the perinatal period and its contribution to distress.

## Appendix

Multimedia Appendix 1 Search string example.

Multimedia Appendix 2 PRISMA (Preferred Reporting Items for Systematic Reviews and Meta-Analyses) checklist.

Multimedia Appendix 3 Quality ratings.

Multimedia Appendix 4 Funnel plot of depression effect size data at the postintervention time point.

Multimedia Appendix 5 Funnel plot of depression effect size data at follow-up.

Multimedia Appendix 6 Funnel plot of anxiety effect size data at the postintervention time point.

Multimedia Appendix 7 Funnel plot of anxiety effect size data at follow-up.

Multimedia Appendix 8 Funnel plot of social tie effect size data at the postintervention time point.

## Abbreviations

CBT: cognitive behavioral therapy
DASS-21: Depression, Anxiety, and Stress Scales–21
EPDS: Edinburgh Postnatal Depression Scale
IPT: interpersonal psychotherapy
PRISMA: Preferred Reporting Items for Systematic Reviews and Meta-Analyses
RCT: randomized controlled trial
SMD: standardized mean difference
