# Supplementary material for: Investigating the Effectiveness of Technology-Based Distal Interventions for Postpartum Depression and Anxiety: Systematic Review and Meta-Analysis
Source: J Med Internet Res. 2024 Nov 19;26:e53236. doi: 10.2196/53236 (PMC11615550; doi:10.2196/53236)
Supplement: Multimedia Appendix 1 [file jmir_v26i1e53236_app1.docx]

**Supplementary Materials**

**Supplementary Material A. Search string example**

"mental health" OR depress* OR anxi* OR stress OR "mental illness" OR wellbeing OR well-being OR psychosocial OR panic OR "mental ill health" OR mood OR trauma OR distress OR "affective disorder" OR "mental disorder"

postnatal OR perinatal OR postpartum OR parent OR birth OR antenatal OR peripartum OR father OR mother OR adopt* OR post-adoption

intervention OR program OR prevention OR psychotherapy OR therapy OR "behaviour therapy" OR "cognitive therapy" OR "behavioural activation" OR "cognitive behaviour" OR cognition OR "cognitive psychotherapy" OR "cognition therapy" OR "behavioral therapy" OR "cognitive arousal" OR "cognitive behavioral" OR "cognitive behavioural" OR CBT OR ICBT OR "cognitive restructuring" OR psychoeducation OR "behaviour modification" OR "cognitive remediation" OR self-help OR exercise OR IPT OR CCBT OR psychotherap* OR therap* OR behavioral activation OR DBT OR "dialectical behaviour therapy" OR ACT OR "acceptance and commitment therapy" OR MBCT OR mindfulness

distal OR remote OR online OR internet OR distance OR e-therapy OR telehealth OR telemedicine OR technology-based OR web-based OR media OR computer-assisted OR mobile OR application OR "mobile application" OR computer OR web*site* e-health OR m-health OR mhealth OR digital OR "phone app*" OR podcast OR website OR "social media" OR phone OR "mobile app*" OR smartphone OR "smartphone tool" OR "mobile health" OR e-book OR SMS OR "text-messag*" OR chatbot

trial OR experiment OR pre-post OR evaluation
