# Supplementary material for: Investigating the Effectiveness of Technology-Based Distal Interventions for Postpartum Depression and Anxiety: Systematic Review and Meta-Analysis
Source: J Med Internet Res. 2024 Nov 19;26:e53236. doi: 10.2196/53236 (PMC11615550; doi:10.2196/53236)
Supplement: Multimedia Appendix 3 [file jmir_v26i1e53236_app3.docx]

**Supplementary Material B. Quality Ratings**

| **Author (year)** | **True randomisation used** | **Group allocation concealed** | **Groups similar at baseline** | **Outcome assessors blinded** | **Groups treated identically other than the intervention** | **Follow-up complete or differences between groups at follow-up described and analyzed** | **Participants analyzed in the groups to which they were randomized** | **Outcomes measured in the same way between groups** | **Outcomes measured reliably** | **Appropriate statistical analysis used** | **Appropriate trial design and any deviation from the standard RCT design accounted for in the analysis** |
| --- | --- | --- | --- | --- | --- | --- | --- | --- | --- | --- | --- |
| Barrera et al., 2015 | √ | √ | √ | NA | √ | Unsure | × | √ | √ | √ | √ |
| Boyd et al., 2019 | √ | √ | × | N/A | Unsure | × | √ | √ | √ | Unsure | √ |
| Carona et al., 2023 | √ | × | √ | N/A | √ | √ | √ | √ | √ | √ | √ |
| Chan et al., 2019 | √ | √ | √ | N/A | √ | √ | √ | √ | √ | √ | √ |
| Danaher et al., 2023 | √ | √ | √ | N/A | √ | √ | √ | √ | √ | √ | √ |
| Fonseca et al., 2020 | √ | √ | × | N/A | √ | × | √ | √ | √ | √ | √ |
| Guo et al., 2020 | √ | √ | √ | N/A | √ | √ | N/A | √ | √ | √ | √ |
| Haga et al., 2019 | √ | √ | √ | N/A | √ | √ | √ | √ | √ | √ | N/A |
| Heller et al., 2020 | √ | √ | √ | N/A | √ | √ | √ | √ | √ | √ | √ |
| Kavanagh et al., 2021 | √ | √ | √ | N/A | √ | √ | √ | √ | √ | √ | √ |
| Lennard et al., 2021 | √ | Unsure | × | N/A | √ | × | √ | √ | √ | √ | √ |
| Loughnan et al., 2019 | √ | × | √ | N/A | √ | √ | √ | √ | √ | √ | √ |
| Nishi et al., 2022 | √ | √ | √ | N/A | √ | × | √ | √ | √ | √ | √ |
| O’Mahen et al., 2013 | √ | × | √ | N/A | √ | × | √ | √ | √ | × | √ |
| Qin et al., 2022 | √ | × | × | N/A | √ | × | Unsure | √ | √ | Unsure | √ |
| Shorey et al., 2017 | √ | √ | √ | N/A | √ | √ | √ | √ | √ | × | √ |
| Sun et al., 2021 | √ | √ | √ | N/A | Unsure | √ | √ | √ | √ | √ | √ |
| Zhang et al., 2023 | √ | √ | √ | N/A | √ | √ | √ | √ | √ | √ | √ |
