# Supplementary figures and images for: Investigating the Effectiveness of Technology-Based Distal Interventions for Postpartum Depression and Anxiety: Systematic Review and Meta-Analysis
Source: J Med Internet Res. 2024 Nov 19;26:e53236. doi: 10.2196/53236 (PMC11615550; doi:10.2196/53236)

**Supplementary Material C. Funnel plot of depression effect size data at post-intervention**


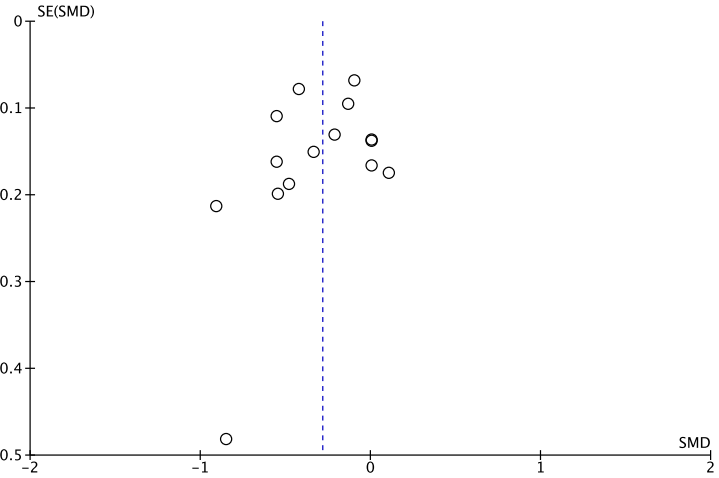

Supplement: Multimedia Appendix 4 [file jmir_v26i1e53236_app4.docx]

**Supplementary Material D. Funnel plot of depression effect size data at follow-up**


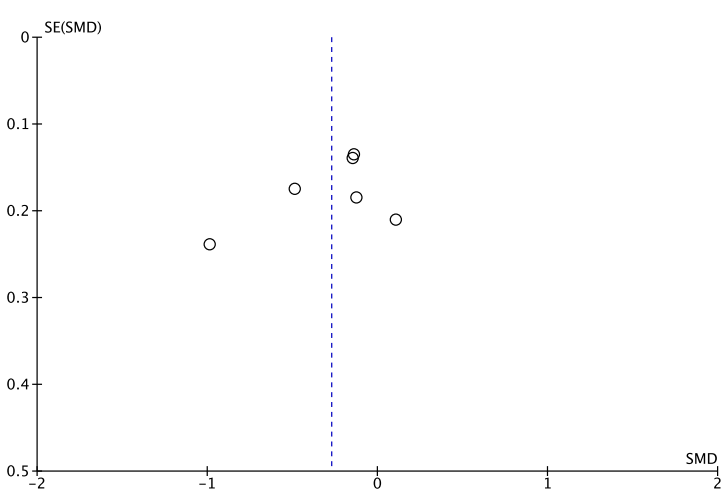

Supplement: Multimedia Appendix 5 [file jmir_v26i1e53236_app5.docx]

**Supplementary Material E. Funnel plot of anxiety effect size data at post-intervention**
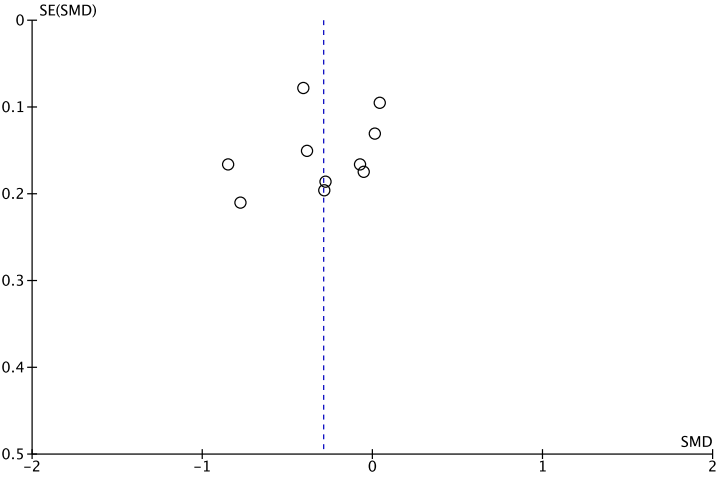

Supplement: Multimedia Appendix 6 [file jmir_v26i1e53236_app6.docx]

**Supplementary Material F. Funnel plot of anxiety effect size data at follow-up**


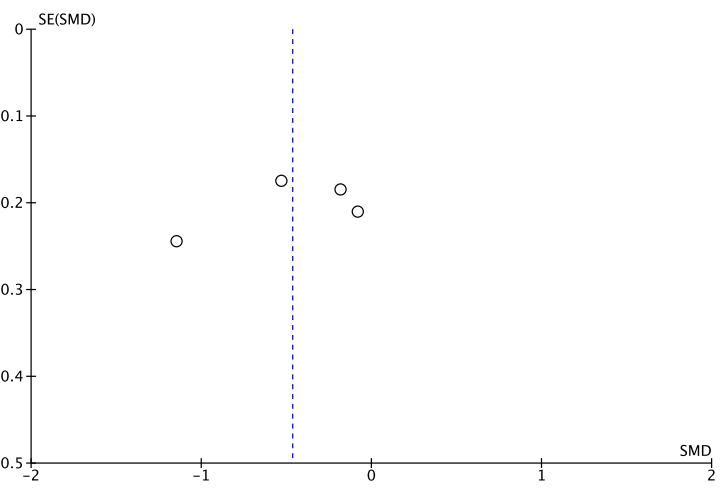

Supplement: Multimedia Appendix 7 [file jmir_v26i1e53236_app7.docx]

**Supplementary Material G. Funnel plot of social ties effect size data at post-intervention**


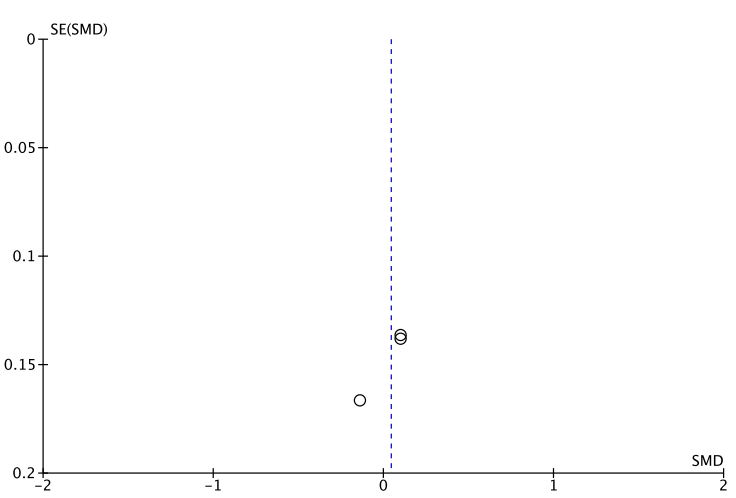

Supplement: Multimedia Appendix 8 [file jmir_v26i1e53236_app8.docx]
